# Accrual Patterns for Clinical Studies Involving Quantitative Imaging: Results of an NCI Quantitative Imaging Network (QIN) Survey

**DOI:** 10.18383/j.tom.2016.00169

**Published:** 2016-12

**Authors:** Brenda F. Kurland, Sameer Aggarwal, Thomas E. Yankeelov, Elizabeth R. Gerstner, James M. Mountz, Hannah M. Linden, Ella F. Jones, Kellie L. Bodeker, John M. Buatti

**Affiliations:** 1 Departments of Biostatistics and; 2 Radiology, University of Pittsburgh, Pittsburgh, Pennsylvania;; 3 Department of Medicine, George Washington University, Washington, D.C.;; 4 Institute for Computational and Engineering Sciences, and Departments of Biomedical Engineering and Internal Medicine, The University of Texas at Austin, Austin, Texas;; 5 Department of Neurology, Massachusetts General Hospital, Boston, Massachusetts; ^5^Department of Radiology, University of Pittsburgh, Pittsburgh, Pennsylvania;; 6 Division of Medical Oncology, University of Washington, Seattle, Washington;; 7 Department of Radiology and Biomedical Imaging, University of California, San Francisco, San Francisco, California; and; 8 Department of Radiation Oncology, University of Iowa, Iowa City, Iowa

**Keywords:** clinical trial, MRI, PET, patient enrollment, accrual

## Abstract

Patient accrual is essential for the success of oncology clinical trials. Recruitment for trials involving the development of quantitative imaging biomarkers may face different challenges than treatment trials. This study surveyed investigators and study personnel for evaluating accrual performance and perceived barriers to accrual and for soliciting solutions to these accrual challenges that are specific to quantitative imaging-based trials. Responses for 25 prospective studies were received from 12 sites. The median percent annual accrual attained was 94.5% (range, 3%–350%). The most commonly selected barrier to recruitment (n = 11/25, 44%) was that “patients decline participation,” followed by “too few eligible patients” (n = 10/25, 40%). In a forced choice for the single greatest recruitment challenge, “too few eligible patients” was the most common response (n = 8/25, 32%). Quantitative analysis and qualitative responses suggested that interactions among institutional, physician, and patient factors contributed to accrual success and challenges. Multidisciplinary collaboration in trial design and execution is essential to accrual success, with attention paid to ensuring and communicating potential trial benefits to enrolled and future patients.

## Introduction

Advances in genomic science and imaging technology have generated an unprecedented level of data analyzed to increase knowledge about the underpinnings of cancer pathogenesis. As a direct consequence, a substantial number of novel targeted agents are being developed and evaluated (1). Within this context, imaging-based biomarkers have well-recognized potential for determining treatment selection and assessing therapeutic response (2). In particular, quantitative imaging biomarkers are anatomically and physiologically relevant numerical features extracted from data within medical images (3). To develop and validate an accurate imaging biomarker for disease diagnosis and prediction of clinical outcome, there is a need for high-quality prospective clinical studies (4). Initiation of these studies requires time-intensive tasks such as preparation of a clinical protocol, obtaining funding for the study, and gaining institutional review board approval. Novel imaging agents require investigational new drug applications; an extensive infrastructure is required to track registration, protocol adherence, and adverse events. Finally, once the funding and regulatory approvals are met, and patient recruitment is initiated, a major barrier to progress is low patient participation rates (5). More specifically, the number of patients enrolling is insufficient for completing active studies; for example, Korn et al. investigated 149 nonpediatric cooperative group phase III trials activated from 2000 to 2007 and estimated that 28% of these trials failed because of low accrual (6). This contribution addresses accrual challenges experienced in cancer clinical trials, specifically involving quantitative imaging, through a survey distributed to institutions actively engaged in these trials.

Several research studies and systematic reviews have identified challenges to cancer clinical trial accrual (7). Highlights of challenges found in these studies include: patients may distrust the design of clinical trials (eg, wary of being placed on the “placebo” arms or how the process of randomization may affect their therapy) or be fearful of side effects from experimental drugs (8, 9). Patients may also be concerned about extra time and travel required for trial participation (10), as well as incremental additional financial expenditures (11). Additional findings show that physician attitudes toward research in general and to specific trials are perceived to affect accrual (12). Referring physicians will lack commitment to study accrual if they perceive institutional barriers related to staffing and strict eligibility requirements (9).

Recruitment challenges for trials involving imaging may differ somewhat from cancer clinical trials as a whole. Patients hear hype about new therapies and genomic targeting in the media, but (despite the aesthetic appeal of radiographic images) advanced imaging has not been touted as widely. Medical oncologists rather than radiologists tend to be principal investigators and approach patients; therefore, trials emphasizing imaging will likely be multidisciplinary and complex. We initiated what is, to the best of our knowledge, the first study to systematically investigate recruitment challenges specific to cancer clinical trials involving quantitative imaging. The present study summarizes results of a survey of physicians, clinical research coordinators, and other professional staff involved in these trials. The survey queried accrual performance and perceived barriers to accrual and solicited solutions to these accrual challenges.

## Methodology

### Survey Instrument Construction

A 35-question survey (included as a Supplemental Appendix) was developed by the Clinical Trial Design and Development Working Group (CTDDWG) of the Quantitative Imaging Network (QIN). The content was developed after a review of the literature cited above, and discussion during CTDDWG monthly teleconferences, followed by a pilot at 4 QIN sites and subsequent modifications. Respondents were invited to fill out 1 survey instrument per study that included novel quantitative imaging biomarkers, or biomarker refinement, as primary or secondary objectives. The survey instrument comprised basic study information, study imaging protocol (including the time requirement for participation), accrual goals, accrual challenges, and perceived solutions for addressing accrual challenges. The basic study information section elicited the following background information about the study including: study site, malignancy, purpose of the study, role of the respondent with respect to the study, and imaging modality(ies) under investigation. The burden of participation for study participants was defined as the number of additional clinic visits and additional hours related to study imaging that were required by the protocol in the first month, and throughout the study. Survey participants were asked to comment on how and/or if patients were compensated for study participation. Accrual goals were assessed through questions concerning total expected enrollment, number enrolled, and expected/actual rate of accrual per year at the time of survey response. Specific accrual challenges were queried. Additional questions assessed the motivation for patients to participate, reasons patients declined participation, and thoughts on strategies to improve accrual. The survey included both single-best answer questions and free text fields.

### Survey Dissemination

The University of Pittsburgh's Institutional Review Board was informed of the project and designated the study as “exempt human subjects research” under section 45 CFR 46.101(b)(2). Survey data were collected and managed using Research Electronic Data Capture (REDCap) tools hosted at the University of Pittsburgh (13). The survey was initially piloted by 4 QIN sites. After further discussions with the CTDDWG, subsequent revisions were made and the content was finalized in early February 2015. The survey instrument was made available via an e-mail invitation that included a URL for a public survey (not linked to the invitation). Sixty-one principal investigators and CTDDWG members from 21 active QIN sites in the USA and 2 international affiliates (All India Institute of Sciences and Tata Memorial Cancer Center) were invited to participate. Recipients were encouraged to forward the invitation to include other sites and research studies involved in clinical research of quantitative imaging. All recipients of the survey were informed regarding the purpose of the study. Because the primary goal of the study was to share strategies to improve accrual, there were no efforts to ensure that the responses were from a representative sample of QIN projects or to have objective responses without the knowledge of others' responses. A total of 3 survey invitations were sent during the survey period, from February 11, 2015 to June 1, 2015.

Interim reports were presented at CTTDWG meetings and at the QIN annual meeting in April 2015. Potential respondents were encouraged to meet with all personnel actively involved in the conduct of the clinical trial (eg, referring and treating physicians, imaging scientists, and clinical research coordinators/associates) for discussion/consensus on responses to each element of the survey.

### Statistical Analyses

The questionnaire was designed to yield both quantitative and qualitative results. The primary quantitative outcome was the percent annual accrual achieved (ie, the actual-to-planned accrual ratio). Occasional recoding of survey responses was required to merge incomplete responses for the same study or to retroactively combine a response category and a common “other” response. For example, for the “perceived reasons for accrual challenges” question, “Scheduling: limited time on clinical scanner” (Supplemental Appendix, page 5) was combined with the “other” response “limited time on research scanner” and reported as “limited time on scanner” (Table 2).

Quantitative and qualitative data were tabulated in aggregate and by subgroups defined by imaging modality. The imaging modality was not designed as a primary covariate of interest, but it served as a useful proxy for burden of participation (which was interpreted somewhat differently among respondents), as research positron emission tomography (PET) scans are more likely than magnetic resonance imaging (MRI) to require an additional clinic visit. Associations between percent annual accrual achieved and potential explanatory variables were explored graphically and summarized using Spearman rank correlation coefficients and the Wilcoxon rank-sum test. Statistical analyses were conducted using SAS/STAT software, version 9.4 (SAS Institute, Inc., Cary, North Carolina) and R version 3.1.3 (R Foundation for Statistical Computing, Vienna, Austria).

## Results

### Study Characteristics

Responses for 25 prospective studies were received from 12 sites (QIN, 10; non-QIN, 2). The following were the primary imaging modalities for these studies: MRI (n = 11 studies), computed tomography (CT; n = 1 study), and PET (including PET/CT and PET/MRI scanners; n = 13 studies).

The total planned recruitment for the 25 prospective trials ranged from 8 to 5000 with a median of 47.5 patients. The median number of patients enrolled was 25, ranging from 1–1118 (Table 1), but most trials were still actively enrolling patients. The median planned annual rate of accrual was 15 patients/year, ranging from 4 to 500. The median actual annual rate of accrual was 11 patients/year, ranging from 1 to 280, with a median actual/planned yearly accrual of 95%.

**Table 1.** Characteristics of Prospective Trials Reported in the QIN Accrual Survey

	MRI or CT (N = 12)n (%)	PET, PET/MRI, or PET and MRI(N = 13)n (%)	Total(N = 25)n (%)
Tumor site			
Brain	4 (33)	2 (15)	6 (24)
Breast	5 (42)	3 (23)	8 (32)
Any solid tumor	—	2 (15)	2 (8)
Head and neck	2 (17)	2 (15)	2 (8)
Pancreas	2 (17)	—	2 (8)
Other^a^	1 (8)	4 (31)	5 (20)
Study types			
Clinical trial of novel therapy	6 (50)	2 (15)	8 (32)
Imaging response	4 (33)	9 (69)	13 (52)
Diagnostic	2 (17)	—	2 (8)
Other^b^	—	2 (15)	2 (8)
	**Median (range)**	**Median (range)**	**Median (range)**
Planned accrual (total)^c^	50.5 (8–800)	45 (20–5000)	45 (8–5000)
Planned minus actual accrual (annual)	0 (−10–17)	3 (−12–220)	0.5 (−12–220)
Percent annual accrual attained^c^	100 (67–350)	75 (3–144)	94.5 (3–350)
No. of study clinic visits (month 1)	0.5 (0–5)	1 (0–3)	1 (0–5)
No. of imaging study hours (month 1)	1.5 (0–8)	4 (0–9)	2 (0–9)
No. of study clinic visits (total)	1 (0–10)	1 (0–5)	1 (0–10)
No. of imaging study hours (total)	2.5 (0–10)	4 (0–15)	3 (0–15)

Abbreviations: MRI, magnetic resonance imaging; CT, computed tomography; PET, positron emission tomography; No., number.

^a^ Lung, pelvic sites, soft tissue sarcoma, and neuroendocrine.

^b^ Reproducibility study, prospective registry.

^c^ Two MRI studies did not report accrual information, and a third did not report annual accrual.

The burden of study participation by time was assessed as a quantitative measure that was hypothesized to be associated with the accrual success. Table 1 describes the additional clinic visits and the number of hours required for study participation, for the first month of the study and in total. As expected, MRI and CT studies (where a novel sequence or processing approach may be added to a routine scan) required less additional time than PET studies (for which scans with a novel tracer often require a half-day separate clinic visit), although monitoring for early response could require additional visits for any modality.

Eight of 25 trials (32%) had quantitative imaging objectives as part of an experimental treatment trial. Patients enrolling in these trials had the incentive of access to a novel therapy. For the remaining 17 trials, 12 investigated the imaging of response to standard therapy options, and the remainder concerned diagnostic imaging, test–retest studies, and other imaging-focused objectives.

Seventeen of 25 study responses provided information on compensation for additional imaging procedures. Five studies (29%) reported no financial compensation for study participation. One study (6%) reported paying expenses and a per-visit stipend. The remaining 11 studies (65%) reported compensation for specific expenses such as parking, meals, and lodging as required.

### Accrual Rate and Associations With Study Characteristics

**Figure 1.**
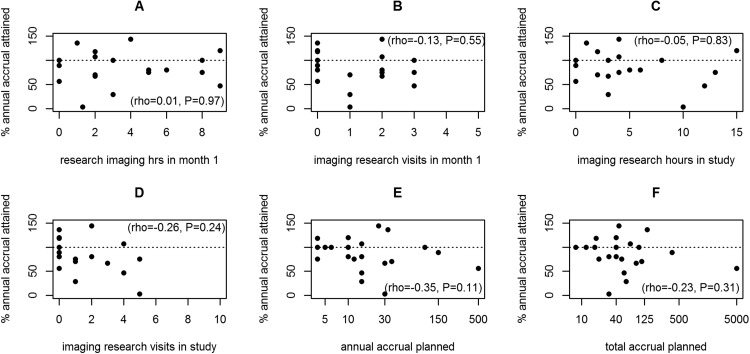
Associations between percent annual accrual attained (100 × Number actual/Number planned) and study burden of participation (A–D) and total enrollment goals (E–F). Spearman correlation coefficient and *P* value are displayed for each panel.

**Figure 2.**
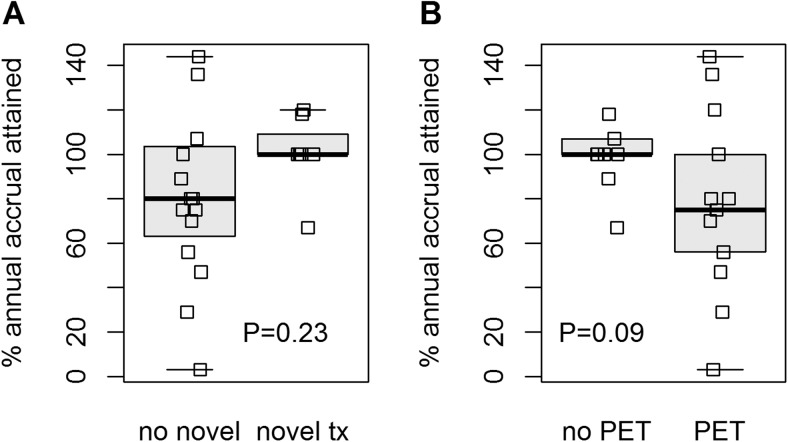
Associations between percent annual accrual attained (100 × Number actual/Number planned) and study characteristics. Quantitative imaging study was or was not part of a clinical trial evaluating a novel therapy (A); imaging modalities examined included PET (PET, PET/CT, and PET/MRI) or did not include PET (MRI, CT) (B). *P* values are displayed from Wilcoxon rank-sum tests.

The primary measure of accrual success was the percent annual accrual (actual/expected), for which sufficient information was provided in 22 of the 25 prospective studies reported. The lowest percent annual accruals were 3%, for a study enrolling 1/30 planned patients in 1 year, and 22/60 for a 4-year study. However, half of the studies reported met or exceeded accrual goals. Figures 1 and 2 show associations between percent annual accrual and potential explanatory variables. The studies that struggled with accrual did not appear to have greater time commitment requirements for subjects. In Figure 1A–D, studies with high and low burden of participation are represented both above and below the horizontal line showing attainment of annual accrual goals (100%), and the rank-order associations measured by Spearman correlation coefficients are low and not statistically significant (*P* > .2). Studies with both high and low annual and total accrual goals also displayed both successful and unsuccessful attainment, without any apparent pattern (Figure 1E–F). In our limited sample, studies involving a novel therapy (a treatment not available outside a clinical trial) tended to have more successful recruitment (median = 100%, 95% confidence interval for mean 85%–117%) than studies without a novel treatment (median = 80%, 95% confidence interval for mean 52%–140%) (Figure 2A; Wilcoxon rank-sum test *P* = .23). Comparing trials with and without PET imaging, those without PET had more successful recruitment (median = 100%, 95% confidence interval for mean 60%–191%) than those with PET (median = 75%, 95% confidence interval for mean 54%–103%; Figure 2B, *P* = .09).

### Qualitative Results

The survey elicited opinions on recruitment challenges and successful strategies through both forced-choice and free response questions (see full survey in Supplemental Appendix). When asked to “check all that apply” for categories of barriers to clinical recruitment (Table 2), the most commonly cited (n = 11/25, 44%) was that “patients decline participation.” Regardless of whether this item was checked, respondents were asked what reasons patients gave for not participating in imaging studies. The most common reasons were “logistical difficulties” (n = 12/25, 48%), “feeling overwhelmed and no extra energy for research” (n = 12/25, 48%), and “[study participation would take] too much time” (n = 8/25, 32%).

**Table 2.** Perceived Accrual Challenges (“Check All that Apply”) Reported in the QIN Accrual Survey

Accrual Challenge	% Reporting (N = 25)
Patients decline participation	44
Too few eligible patients	40
Potentially eligible patients are not approached	32
Limited time on scanner	24
Competing trials do not allow co-enrollment	24
Scheduling: imaging staff not available when patient is	16
Scheduling: scanner broken/tracer unavailable	8
Other	28
Lack of funding for adequate staffing (3)	
Limited time on research scanner (1)	
Trial participation may interfere w/insurance (1)	
Data exchange challenges (1)	
Difficult to schedule imaging within treatment time constraints (1)	

The second most common (n = 10/25, 40%) accrual challenge was “too few eligible patients.” This limitation was noted even for studies that achieved their accrual goals. The next most frequently selected challenge was that “potentially eligible patients are not approached” (n = 8/25, 32%). Prompted for the reason patients were not approached (check all that apply), the most common response was “referring oncologist/surgeon not enthusiastic about study” (n = 4/8), followed by “competing trial higher institutional priority” (n = 3/8) and, 2 “other” responses describing misunderstanding of eligibility by research staff.

A separate question allowed only 1 response: “What is your single greatest recruitment challenge for this study?” (Table 3). For 8/25 studies (32%), “too few eligible patients” was cited, and 1 “other” response noted that co-enrollment in a phase 1 study was dependent on timing for opening and closing dose cohorts. The next most common reason given (n = 4/25, 16%) was “patients are reluctant—study takes too much time,” with an additional response that patient reluctance was a challenge, but study staff were not certain of the reasons for patient reluctance. When asked about accrual from a positive point of view (reasons patients give for participating in research studies involving quantitative imaging biomarkers), respondents for most studies cited “contribute to cancer research” (n = 22/25, 88%), and often felt that patients wanted to “find out more about their own cancer” (n = 14/25, 56%), or participated because their “physician recommended the study” (n = 13/25, 52%). Hope for therapeutic benefit was cited as an “other” reason for 2 studies from the same site. In contrast, there were no prompted resources that respondents agreed would be useful for overcoming enrollment challenges. “Simplifying the imaging protocol” was identified for 4/25 studies (16%). “Broader patient eligibility,” “resources for patients,” and “resources for colleagues” were each identified for 3/25 (12%) of responses.

**Table 3.** Perceived Greatest Accrual Challenge Reported in the QIN Accrual Survey

Accrual Challenge	% Reporting (N = 25)
Too few eligible patients	32
Patients are reluctant—study takes too much time	16
Difficulty with availability of research staff due to understaffing/turnover	12
Patients are reluctant—study does not benefit them personally	8
Potentially eligible patients are put on competing trials without co-enrollment	4
No response	8
Other	20
Unspecified patient reluctance (1)	
Enrollment limitations of (phase 1) parent study (1)	
Scheduling difficulties (1)	
No challenges (2)	

## Discussion

Clinical trials are essential for advancing oncology care. In addition to the labor-intensive tasks required for trial preparation and approval, patient accrual is extremely important for trial success. This study is the first to examine factors affecting accrual in clinical trials involving quantitative imaging.

Half of the studies reported meeting or exceeding their annual accrual goals. Only 3/22 (14%) were accruing at less than half the rate planned, a benchmark for triggering a formal review of studies sponsored by National Cancer Institute (NCI)'s Cancer Therapy Evaluation Program. While this is similar to cooperative group oncology trials (14), selective reporting of quantitative imaging trials that had better accrual may have occurred. In addition, a group heavily invested in quantitative imaging research (ie, the NCI's QIN) may have the expertise required for optimizing the chances of successful accrual.

We found that only 1/7 trials featuring novel therapies failed to meet or exceed annual accrual goals, compared with 10/15 studies without a novel therapy (Figure 2A). Novel therapies may be a powerful incentive for clinical trial participation. For example, an analysis of 787 phase II/III NCI Cooperative Group Clinical trials found that studies evaluating a new investigational agent or a targeted therapy were more likely to have successful accrual (14).

Studies involving MRI or CT without PET tended to have better accrual success than studies involving PET (Figure 2B). Studies with standard and novel PET tracers have several of the following features that may limit accrual success: a large time burden for novel imaging procedures, patient preparation burden (such as fasting for 18F-fluorodeoxyglucose-PET), and concern regarding additional radiation exposure. A typical body survey, in addition to the radio-tracing agent used, exposes the patient to an effective dose up to 18.5 MBq (15), half the annual occupational dose limits for adults (37 MBq). Although the radiation risk is real, long-term risk for future malignancy must be weighed against potential benefit for optimal treatment of an existing malignancy. Furthermore, recent developments in technology may reduce PET/CT radiation dose (16, 17), and the development of combination scanners (ie, PET/MRI) may help address both the logistical burden of scheduling research scans and the burden of participation for research participants.

Accrual was not shown to be related to the amount of time required for research imaging. However, the time burden of research participation was listed as a frequent reason that survey participants believe patients decline participation. Common accrual challenges other than patients declining participation included too few patients meeting trial eligibility and that potentially eligible patients were not approached. Follow-up questions cited lack of referring physician's enthusiasm for the study (n = 4/8 indicating that patients were not approached, 50%) and competing trials of higher institutional priority (n = 3/8, 38%) as common reasons potentially eligible patients were not approached. These competing trials do not necessarily address the most pressing questions in oncology practice, but it may reflect the priorities of pharmaceutical companies and junior researchers under pressure to produce results quickly (18). Studies in NCI-designated cancer centers prioritize participation in therapeutic trials as a metric of success and de-emphasize “nontherapeutic” trials that may define many quantitative imaging trials. Institutional factors such as study prioritization and lack of access to scanners and study personnel suggest that all these factors interact to deter physicians from approaching patients about quantitative imaging-based clinical trials.

There are some limitations to our study. The 25 quantitative imaging studies with survey responses represent a snapshot of trials from a select number of institutions. These institutions were identified because of their interest in quantitative imaging so reflect a motivated set of investigators where accrual may be higher than elsewhere: the reality of accrual in quantitative imaging trials may be worse than we report. We did not directly query patients, who may have offered additional insights into barriers for participation. We also did not measure certain factors that are known to be associated with accrual success, such as trial development time (19). Finally, most trials were actively accruing; clinical trial enrollment often starts slowly as the kinks of enrollment are worked out. Therefore, the measure of percent annual accrual may not accurately reflect ultimate accrual success for trials in progress.

In conclusion, evidence-based strategies for improving oncology clinical trial accrual are sparse (7). A national probability sample survey of patient attitudes toward oncology clinical trial participation suggested much more willingness to participate than that represented in actual accrual (20). The results from the present study, taken together with existing literature on clinical trials in oncology, suggest that clinical trials to develop imaging biomarkers to improve cancer diagnosis and treatment can be improved by:

(1) Integrating quantitative imaging questions into therapeutic trials.
(2) Simplifying imaging protocols and trial eligibility to enable more rapid accrual in high-impact areas.
(3) Better recognition and accommodation of study participants' contributions, perhaps including monetary compensation.
(4) Clear language as part of informed consent to explain how the trial results may benefit the cancer community and individual patient.
(5) Effective outreach to clinicians concerning the development/validation of imaging biomarkers as a tool for diagnosis, staging, and prediction of cancer and survival outcome.

In agreement with studies querying patients directly, our respondents reported that patients cite “physician recommendation” as a strong reason for considering trial participation. Hence, including the treating oncologists in the design of the imaging components of the trial and integration into their most important questions of interest is likely to be important. Quantitative imaging studies, like other precision medicine initiatives with a strong biomarker component, require multidisciplinary collaboration and enthusiasm from all these disciplines to ensure study success.

### Supplemental Materials

Supplemental Appendix:
