# Supplementary material for: Accrual Patterns for Clinical Studies Involving Quantitative Imaging: Results of an NCI Quantitative Imaging Network (QIN) Survey
Source: Tomography. 2016 Dec;2(4):276–82. doi: 10.18383/j.tom.2016.00169 (PMC5260812; doi:10.18383/j.tom.2016.00169)
Supplement: Supplemental Appendix: [file tom-00169-16-s001.pdf]

Accrual Survey: Clinical studies with Quantitative Imaging Biomarkers

Please complete the survey below.

There is a link to the study FAQ at the top of each page. Please contact bfk10@pitt.edu and saggarwal@gwmail.gwu.edu with further questions.

Thank you!

Basic Study Information

(FAQ for Accrual Questionnaire)

QIN site

- ◯ Not a QIN site or affiliate
- ◯ Univ Iowa
- ◯ Univ Pittsburgh
- ◯ Stanford
- ◯ Vanderbilt
- ◯ Moffitt
- ◯ Univ Washington
- ◯ Brigham and Women's
- ◯ Mass General
- ◯ Columbia
- ◯ Oregon Health & Science
- ◯ Johns Hopkins
- ◯ UCSF
- ◯ MSKCC
- ◯ Univ Michigan
- ◯ Mayo
- ◯ Mt Sinai
- ◯ Emory
- ◯ Washington Univ
- ◯ UCLA
- ◯ Med Coll of Wisc
- ◯ AIIMS - India
- ◯ TMC - India
- ◯ Other

Name of other site ________________________________

Full title of study ________________________________

Abbreviated title of study ________________________________
(The nickname you use, like SWOG 0048 or p13-246)

Is the study conducted at multiple sites?

- ◯ Single site
- ◯ Multi-center

(regional practice network is still single site)

Cancer Site

- ◯ Brain
- ◯ Head & neck
- ◯ Lung
- ◯ Breast
- ◯ Prostate
- ◯ Any solid tumor
- ◯ Other

Other cancer site ________________________________

Type of study (primary reason patients enroll)

- ◯ Therapy trial (novel therapy - only available on study)
- ◯ Imaging of response to therapy (not a therapy trial)
- ◯ Repeatability/reproducibility imaging trial
- ◯ Other

(for RETROSPECTIVE STUDIES choose other and choose "imaging study is a retrospective analysis" as QI/QIN role below)

Other study type __________________________________

Imaging modality

- ☐ PET
- ☐ MR
- ☐ CT
- ☐ Ultrasound
- ☐ other

((check all that apply))

Imaging modality (other) __________________________________

Quantitative Imaging (QI) and QIN role in study

- ◯ Primary study aims are imaging (QIN study)
- ◯ Primary study aims are imaging (non-QIN study)
- ◯ Imaging correlative study - separate enrollment from a parent study (QIN study)
- ◯ Imaging correlative study - separate enrollment from a parent study (non-QIN study)
- ◯ Prospective analysis of images from a parent study (scanner time not funded by imaging study)
- ◯ Imaging study is a retrospective analysis
- ◯ other

Other QIN role __________________________________

Your Name __________________________________

Your Role in the QIN study __________________________________

(research nurse, research coordinator, doctoral student research assistant, PI.... If completing survey as a team, say "team" and describe in comments below)

Your preferred contact information __________________________________

(email address or phone number or supervisor's email...)

Comments about basic study information __________________________________

Study imaging protocol (beyond standard of care)

(FAQ for Accrual Questionnaire)

Number of additional clinic visits required for study imaging: month 1 of protocol
____
(excluding standard of care imaging, or scans/sequences performed in the same visit as standard of care)

Number of additional hours required for study imaging: month 1 of protocol
____
(may be fraction of an hour for additional sequences, or full time in clinic for additional visit (ie, FDG PET))

Number of additional clinic visits required for study imaging: full protocol
____
(excluding standard of care imaging, or scans/sequences performed in the same visit as standard of care; including month 1)

Number of additional hours required for study imaging: full protocol
____
(may be fraction of an hour for additional sequences, or full time in clinic for additional visit (ie, FDG PET); including month 1)

How are patients compensated for their time spent in additional imaging procedures?
- ◯ Expenses only (< $25/visit)
- ◯ Expenses plus per-visit stipend
- ◯ Stipend only (>= $25/visit)
- ◯ Other

Other compensation
____

Comments about study demands on patient time versus compensation
____

Accrual goals

(FAQ for Accrual Questionnaire)

Total Study Enrollment planned in protocol

____

(respond for your site only in a multicenter study, unless you are the coordinating site with direct knowledge to answer the remaining questions in this section)

Date study was open to accrual

____

((enter 1 if uncertain of day of the month))

Number of patients enrolled (and contributing at least some imaging study data)

____

Number of patients who were engaged in the process of informed consent, and who declined participation

- ◯ < 1 per patient enrolled
- ◯ 1-2 per patient enrolled
- ◯ >2 per patient enrolled

Comments about patients who declined to enroll after being approached

____

Number of patients approached and found ineligible

- ◯ < 1 per patient enrolled
- ◯ 1-2 per patient enrolled
- ◯ >2 per patient enrolled

Comments about ineligible patients

____

Expected rate of accrual stated in protocol

____

(per year)

Actual rate of accrual so far

____

(per year --- results will only be reported in aggregate!)

Other comments about accrual goals

____

Accrual Challenges

(FAQ for Accrual Questionnaire)

Perceived reasons for accrual challenges

- ☐ Too few eligible patients
- ☐ Competing trials do not allow co-enrollment
- ☐ Potentially eligible patients are not approached
- ☐ Patients decline participation
- ☐ Scheduling: limited time on clinical scanner
- ☐ Scheduling: scanner broken/tracer unavailable
- ☐ Scheduling: imaging staff not available when patient is
- ☐ Other

((check all that apply))

Other perceived reasons for accrual challenges __________________________________

Why do you think patients were not approached

- ☐ QIN staff not available when primary study contacted to see a patient
- ☐ Gaps in QIN research staffing due to turnover
- ☐ Referring oncologist / surgeon not enthusiastic about QIN study
- ☐ A competing trial is higher institutional priority
- ☐ Other

((check all that apply))

Other reasons patients were not approached for the QIN study __________________________________

Why do patients tell you they refuse participation in this imaging study

- ☐ Too much time
- ☐ Logistical difficulties
- ☐ Feeling overwhelmed and no extra energy for research
- ☐ Worried about radiation exposure
- ☐ Chose a competing trial
- ☐ Other

((check any that apply for this study))

Other reasons patients refused this imaging study __________________________________

Why do patients tell you they do participate in this QIN study

- ☐ Contribute to cancer research
- ☐ Find out more about their own cancer
- ☐ Physician recommended the study
- ☐ Other

((check any that apply for this study))

Other reasons patients participate in this QIN study __________________________________

What is your single greatest accrual challenge for this study?

- ◯ Too few eligible patients
- ◯ Potentially eligible patients are put on competing trials without co-enrollment
- ◯ Referring physicians do not recommend the study to patients
- ◯ Difficulty with availability of QIN research staff due to understaffing/turnover
- ◯ Patients are reluctant - study takes too much time
- ◯ Patients are reluctant - study does not benefit them personally
- ◯ Patients are reluctant - fears about radiation exposure
- ◯ Other

Greatest challenge to accrual (other) __________________________________

Accrual challenges comments __________________________________

Addressing accrual challenges

(FAQ for Accrual Questionnaire)

What has helped you to address accrual challenges? __________________________________

What might help?
- ☐ Resources for colleagues
- ☐ Resources for patients
- ☐ Other resources
- ☐ Simplify imaging protocol
- ☐ Broader patient eligibility

Description of suggested helpful resources __________________________________

What challenges have you faced in acquiring images for retrospective studies? __________________________________
(IRB hurdles, technical difficulty with deidentification, etc)

What resources are helpful for acquiring images for retrospective studies? __________________________________
(anonymized images available from other studies, etc)

Other comments __________________________________
